# Research on the Evolution of the Express Packaging Recycling Strategy, Considering Government Subsidies and Synergy Benefits

**DOI:** 10.3390/ijerph18031144

**Published:** 2021-01-28

**Authors:** Yanlu Guo, Gongli Luo, Guisheng Hou

**Affiliations:** College of Economics and Management, Shandong University of Science and Technology, Qingdao 266590, China; luogongli1112@126.com (G.L.); houguisheng001@163.com (G.H.)

**Keywords:** express packaging, heterogeneous parties, government subsidies, evolutionary game

## Abstract

With the year-on-year growth of e-commerce transactions and the increasing popularity of the concept of ecological civilization, the waste and recycling of express packages have aroused widespread discussion and attention. On the issue of express package recycling, how consumers, e-commerce enterprises, and e-commerce platforms choose their own strategies, how to better promote the recycling of express packages, and what is the effect mechanism of government subsidies on different players. These are the questions that this article wants to answer. Since this article involves many stakeholders, in order to better identify the strategic choice and evolution of different entities and to better study the influence of government subsidies on the strategic choice of game players, this article uses two triparty evolutionary game models. The results show that without subsidies, changes in the rate of return and the initial probability will affect the evolution of the equilibrium strategy, while the synergistic benefits will have a corrective effect in some cases; when government subsidies are included and the probability of the three parties choosing “green strategies” is relatively low, subsidies should be paid to e-commerce companies mainly; lower subsidies can only provide incentives for e-commerce platforms. This article can provide certain references and value for government policymakers.

## 1. Introduction

The development of e-commerce has brought great convenience to people’s lives; more and more people tend to choose online shopping. In order to better protect the items purchased by consumers, using different kinds of materials during the process of packaging has become an inevitable choice. Imagine a typical situation: first, you open the thick plastic tape of the express package and open the corrugated cardboard box specially customized by the merchant. Second, you open the plastic antifall bubble film, even some paper filaments and sponges; finally, you take out the items bought online. These packaging materials complete their mission, and you discard them. Such scenes are ongoing. The problems of excessive packaging waste in express delivery have become more and more serious. Zhao and Sun pointed out that since 1978, China has formulated a series of environmental regulations to realize the governance of water resources, soil, air, and other resources [1]. Now the recovery and recycling of express packaging waste have also attracted the attention of policymakers. In 2019, the China News Agency (a Chinese official media) released a report saying that China’s e-commerce transactions in 2018 totaled 31.63 trillion yuan, with an annual rate of increase of 8.5%. The total volume of express business reached 50.71 billion, with an annual rate of increase of 26.6% (here, the data comes from the China E-commerce Development Report 2018–2019. On 8 September 2019, the 2019 Global E-Commerce Conference was held in Xiamen, the China E-commerce Development Report 2018–2019 was also released at the conference. On 13 February 2017, the State Post Bureau issued the 13th Five-Year Plan for the Development of the Express Industry to guide the development direction and path of the express industry; at the same time, the plan also disclosed some development data related to the express delivery industry). According to the 13th Five-Year Plan of the express business industry, the total number of express parcels will reach 70 billion in 2020, and the number of express deliveries per capita will rise from 0.01 in 2000 to 50 by 2020. In order to prevent the items purchased online from being damaged during conveyance, different kinds of packaging materials have been developed and used. However, as the volume of express delivery business increases year by year, the harm of express packaging waste to the environment has gradually attracted people’s attention. People have started to search for more ecological packaging materials and explore new ways of recycling express packages. However, as far as the current situation is concerned, the recycling rate of express packages is still relatively low. People’s Daily (a Chinese official media) pointed out that it is estimated that the current overall recycling rate of express package waste in China is less than 10%. Concretely speaking, the carton recycling rate is less than 20%; the filler and tape recycling rates are even worse close to 0 (People’s Daily was published on 8 December 2017, with the original title of “How far does green express go”, http://finance.people.com.cn/n1/2017/1208/c1004-29693388.html).

In response to these problems, the State Post Bureau (the relevant agency in charge of the express delivery industry in China) issued a plan to promote the express package recycling efforts of the express delivery industry in 2016. This plan aimed to build an express package recycling system by 2020. Giant domestic e-commerce platforms such as Taobao, JD.com, and Suning.com also introduced relevant policies and practices for the recycling of express packages. However, limited by consumers’ environmental awareness and recycling costs, the downstream recycling and reuse rates of express packages are still very poor. Hence, this research mainly answers the following questions:

① What are the key factors that influence different entities to promote express package recycling, and how will the strategic choices of different players affect the evolution of the system as a whole?
② Can government subsidies effectively encourage subjects to choose to participate in express package recycling, and are there differences in incentive power for different subjects? How should policymakers set their own incentive priorities?

As we know, the recycling of express packages involves many actors, such as consumers, e-commerce enterprises, and e-commerce platforms. The triparty evolutionary game model can better handle situations with more participants. There are studies [2,3,4,5] that have used the triparty evolutionary game model to study both strategy selection and evolution in a multiplayer situation. Zhe Wang et al. studied the recycling of e-waste with the participation of multiple entities [4]. Haiyan Shan and Junliang Yang used a triparty evolutionary game model to study the strategic choice and evolution of enterprises, poor households, and governments when studying photovoltaic poverty reduction [6]. At the same time, the model in this article can better discover the incentive effect of government subsidies on different subjects.

In summary, the innovation lies in this article is using two triparty evolutionary game models to discuss the evolution of the strategy of e-commerce platforms, e-commerce companies, and consumers. We believe that when e-commerce companies and consumers have the same strategic preference for express packages, there will be synergy benefits between the two parties. Secondly, this paper considers the evolution of strategies when including and not including government subsidies. In addition, numerical simulations and real cases (the specific practices of Chinese e-commerce giants in express delivery packaging recycling) are combined to explain the results of the previous theoretical model. The article provides enlightenment and guidance for decision-makers in express package recycling.

The rest of this article will be arranged in this way: Section 2 is a literature review; Section 3 introduces the model of express package recycling, considers the situation with and without government subsidies, and briefly analyzes the strategic choices and evolution of the different parties. Section 4 gives numerical simulation examples, subsequent discussion and case studies are given in Section 5, and Section 6 presents the research conclusions, research limitations, and future research directions of this article.

## 2. Literature Review

### 2.1. Packaging Materials or Design, Recycling System, and Recycling Model

Many scholars have studied this topic from the perspective of package design, recycling system, and recycling model. These studies have shed some light on the issue of package recycling. Kondo et al. studied the recycling of various packages from the perspective of the life cycle [7]. Duhaime et al. studied the issue of recyclable packaging utilization by Canada Post and other large mail customers [8]. Ross and Evans et al. studied the recycling strategy of plastic packaging [9], while Pati et al. used the goal planning model to study the recycling of paper packaging [10]. However, these documents only study the recycling problem of specific materials. There is also some literature on the greening and recycling of packaging from the perspective of a reverse supply chain [11,12,13]. Klaiman et al., using discrete experimental research, found that consumers’ willingness to pay for packaging materials is positively related to the recyclability of packaging materials, among which consumers with the highest willingness to pay tend to pay for plastic materials [14]. In terms of express packages, paper materials and plastic materials are the two most widely used materials, both of which have lower cost and plasticity. With the concept of a sharing economy gradually gaining popularity, some studies have begun to conduct research and analysis from the perspective of shared express packaging. Leite pointed out that recyclable packaging has good product protection, cost reduction, and environmental benefits [15]. Silvas et al. demonstrated the benefits of shared packaging using cases [16]. However, there are some documents that point out the disadvantages of shared packaging, such as higher transportation costs, cleaning and maintenance, storage, and capital investment [17], in addition to the costs caused by loss and misplacement [18]. Nevertheless, the overall benefits of packaging recycling are greater than its disadvantages [15]. Twede and Clarke even pointed out that shared packaging is a global trend [19].

### 2.2. Division of Responsibilities for Solid Waste and Its Recycling Process

There are also some studies focusing on the division of responsibility for the recycling or disposal of packaging waste. For example, Sujit Das summarized the three forms—recycling by manufacturers, independent recycling by manufacturers, and third-party recycling from different recycling entities [20]. Stuart Ross summarized the practice and experience of waste treatment in the UK and summarized the British government’s management policies into three types: sustainable development strategy, waste recycling, and environmental protection action [9]. Additionally, he pointed out that, in practice, some policy guidance can be used to encourage enterprises and people to actively participate in recycling. Mohamed Alwaeli studied the impact of product charging policies and relevant EU directives on the level of recycling of waste packaging in Poland [21]. They all pointed out the important role of relevant government policies in promoting packaging recycling and, at the same time, guiding enterprises and individuals to actively participate in it. After studying the relevant recycling policies, Wilmshurst et al. proposed an extension of the production responsibility system to “those who produce the waste being responsible for the waste” and applied this approach to the formulation of packaging waste recycling policies [22]. The extended producer responsibility (EPR) gives us more mature guidance on the collection and disposal of used products [23,24]. Susan E. M. Selki pointed out that the design of express package systems should be taken into account and also facilitate logistics distribution [25]. This view is more from the perspective of the entire chain and process in order to study the issue of package recycling. Nuno Ferreira da Cruz and Sandra Ferreira studied the recycling plans of European countries such as Germany, Fafa, and Portugal and proposed that the governments and industry, under the extended production responsibility system of industry, should coordinate the interests of the entities [26]. Levine D. K., Modica S., and Weinschelbaum F. et al. analyzed the financial problems of packaging waste from the perspective of governments [27]. As a kind of solid waste, there are also documents related to urban solid waste that should attract our attention [28,29]. Pitchayanin Sukholthaman and Alice Sharp summarized the types and hazards of municipal solid waste and analyzed the effectiveness of the current measures [30]. The article pointed out that the treatment of waste should be led by the government; the public and other institutions taking part are the supplementary actors. Liu H. et al. used the grey-DEMATEL method to study the key factors affecting the recycling of construction waste in China [31]. Long H. et al. studied the recycling of construction waste under the consideration of green development performance and the governmental reward-and-punishment mechanism. The study found that the governmental reward-and-punishment mechanism can effectively restrain the production and recycling process [32].

### 2.3. The Application of the Evolutionary Game Model and Its Applicability to This Research

Khan F. et al. studied the behavioral intentions and common practices of consumers in developing countries when dealing with plastic waste [33]. Considering that the main parties and factors involved in express package recycling are relatively complex, this paper uses a three-party evolutionary game model to study the strategy selection, influencing factors, and equilibrium evolution of e-commerce enterprises, e-commerce platforms, and consumers. The evolutionary game method is a classic method that combines biological evolution and game theory technology, and it is widely used in economic and management issues [34,35]. For example, the classic literature of Friedman used this method to study the influence of trade on the organizational form of enterprises [36]. David K. Levine et al. studied the imitation behavior in the cooperation process and concluded that when the imitation behavior of one of the cooperating parties occurs, new problems such as free-riding will appear in the cooperation process [37]. Barari and Agarwal studied the construction of a green supply chain selection model based on the evolutionary game method. The results show that middlemen and producers can achieve maximum supply chain benefits through strategic adjustments [38]. Shen et al. used an evolutionary game model to analyze the recycling of used building materials by contractors and building material manufacturers [6]. Dao zhi Zhao et al. used the three-party evolutionary game model to study the low-carbon capacity sharing problem [39]. Kai Yu et al. used the evolutionary game model and system dynamics methods to study the symmetry of employee behavior in coal enterprises [40]. Qing yun Pang and Mu Zhang used the evolutionary game model to study land income distribution in tourism development [41]. Mengjie You et al. studied the internal safety inspection and regulating policy of Chinese coal companies [42]. Herui Cui et al. used a triparty evolutionary game model to study the sustainable development of green finance [43]. The study found that strengthening government supervision and reducing the cost of green finance for green financial institutions and enterprises can contribute to the ideal evolutionary goal. When studying the promotion and application of solar panels, Yujuan Fang et al. used a triparty evolutionary game model to study the strategic choice and evolution of different entities and found that appropriate subsidies and pricing strategies can effectively promote the goal of cooperation [44]. The emergence and development of the concept of express packaging waste are closely related to the development of e-commerce and logistics systems. Therefore, express packaging waste can be recycled with the help of logistics and e-commerce platforms. The platforms have certain advantages in the delivery of express information and logistics information. On the other hand, express packaging recycling involves more parties than traditional solid waste from a common perspective. Specifically, express packaging recycling needs to involve consumers, e-commerce companies (manufacturing or retail), logistics companies, and e-commerce platforms. How to identify some key factors, with the participation of different parties, to jointly promote the perfection and benign cooperation of express packaging recycling is a problem worthy of attention. Existing literature has pointed out that the government has made some special efforts to promote relevant environmental policies and encourage green behavior [45,46,47], so this article also adds this factor to the model.

## 3. Model

### 3.1. Theoretical Basis

#### 3.1.1. Concept Definition

Although the literature on traditional solid waste recovery and recycling can provide some insights to the research of this article, the recycling of express packaging has some peculiarities. First, express packaging involves more stakeholders than general solid-waste-related research, such as consumers, e-commerce platforms, e-commerce companies, and government policymakers. The increase in the amount of express packaging waste is closely related to the transaction volume of the e-commerce market. In addition, it is worth noting that the concept of “green express packaging” in this article is different from packaging development and production processes in the field of industrial design and materials; it refers to the “reuse of express packaging and a series of actions related to the process of the “selection of ecological materials” that can help reduce express packaging waste. In the existing literature, some studies have studied the treatment of solid waste from the perspective of the extended production responsibility system [2] and the life cycle of packaging materials. As far as the production responsibility extension system is concerned, the division of responsibilities starts from the producer and then extends to the entire life cycle of the product. This approach is suitable for relatively mature large-scale supply chains. As far as express packaging is concerned, it is difficult to attribute the responsibility to the producer due to cost constraints. First of all, consumers can choose whether to participate in the packaging recycling program and can shop at e-commerce merchants that use (or do not use) recyclable express packaging materials. For merchants, they may choose to use or not use recyclable materials to protect their goods. As for the platforms, they can promote express packaging recycling through publicity and other means. Government policymakers play an administrative role and can promote the active participation of different actors in express packaging recycling through certain policies or subsidies. Therefore, starting from the actual situation and existing literature, this article studies the strategic choice and evolution of different stakeholders on this issue.

#### 3.1.2. Problem Description

Since e-commerce platforms (EPs) play a central role in the e-commerce business, the importance of the e-commerce platform in the selection and evolution of express package recycling strategies cannot be ignored. E-commerce retail enterprises (EEs) and consumers (e-commerce consumers (ECs)) are directly affected by the relevant policies. For example, the recycling of express packaging and the use of express package materials will undoubtedly affect the costs of e-commerce enterprises, and these costs will be transferred to consumers in some way, so, in the evolution of express packaging recycling strategies, these three parties are worthy of attention. The government as a policymaker can influence the strategic evolution of these three parties. For actions that are beneficial to environmental protection, the government can use subsidies to encourage and support environmental-friendly strategy, so this paper considers the evolution of the three-party strategy in the two types of situations—with or without government subsidies—to provide some guidance to the relevant policymakers. In the following content, the Y-Model means with government subsidies and the N-Model means without government subsidies to the three parties.

### 3.2. Hypothesis

**Figure 1:**
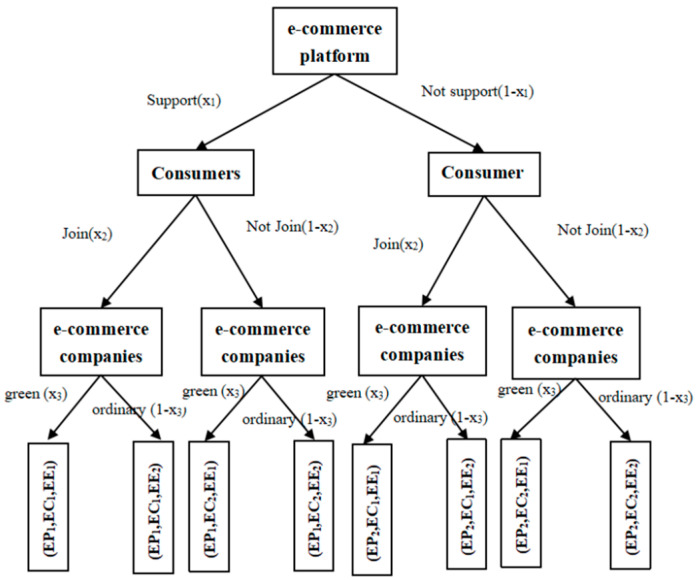
The evolutionary game structure tree of strategies.

Assumption 1: Suppose there are three types of game participants: e-commerce platforms, e-commerce consumers, and e-commerce companies. Express packaging is not simply equivalent to repeatable and recyclable packaging materials but a more complex proposition. Different from the research on packaging materials from the perspectives of a life cycle in industrial design and materials, the concept of an “express package” in this article mainly refers to the ecofriendly behaviors related to express packaging, such as the use of recyclable materials by merchants or platforms. Moreover, consumers, merchants, and platforms choose to use recyclable express materials and actively participate in a series of actions such as packaging recycling. Assuming that the strategy of the e-commerce platform (EP) to promote and support an express package recycling strategy is marked as EP_1_ and not to promote the express package recycling strategy is marked as EP_2_, the probabilities of these two strategies are respectively recorded as $x_{1},1-x_{1},x_{1}\in (0,1)$. Furthermore, e-commerce consumers (ECs) have two types of strategy: join the express package recycling plan (EC_1_) and not join the express package recycling plan (EC_2_); the probabilities of these two strategies are respectively recorded as $x_{2},1-x_{2},x_{2}\in (0,1)$. E-commerce companies (EE) has two types of strategy: choose green express packaging (EE_1_) or choose ordinary packaging (EE_2_); the probabilities of these two strategies are respectively recorded as $x_{3},1-x_{3},x_{3}\in (0,1)$. The three actors are all assumed to be rational, and they modify their strategic choices according to the actual changing situation [35,36]. The evolutionary game structure tree of strategies is shown in Figure 1.

Assumption 2: He et al. found that the main factors affecting whether a company chooses green production include government regulation, indirect benefits, and green costs [48]. The cost of the EP to choose to promote and support the express package recycling strategy is $C_{1}$; the cost to choose not to promote the express package recycling strategy is $C_{2}$. The cost of choosing not to promote an express package recycling strategy is getting just a basic income $R_{0}$. When EPs choose the support strategy, EPs can get a good reputation A. When ECs choose to join the express package recycling plan, they need to pay for more energy and time (remember this cost as $L_{1}$); at this time, ECs can get the utility satisfaction of shopping and the total benefit of psychological satisfaction brought by environmental protection $B_{1}$. When the choice is not to join, ECs only need to pay the basic cost of shopping, marked as $L_{0}$. At this time, ECs only get the satisfaction of shopping, marked as $B_{0}$. When the EP chooses the support strategy, the environmental-friendly behavior will bring greater satisfaction to consumers, and ECs can get greater satisfaction by choosing express package e-commerce companies. More recommendations and exposure will bring additional revenue to e-commerce companies and consumers in this case (remember this additional revenue as $D_{1},D_{2}$). When the EP chooses not to support recycling, no additional revenue will be made. When an EE chooses an express package, it defaults to join the packaging recycling plan, remembering that the cost of selecting express packages is $W_{1}$, the cost of selecting ordinary packaging is marked $W_{2}$. When choosing express packages, in addition to the normal sales revenue, it can convey positive corporate fame and enhance the reputation of the company (mark this part of the revenue as $V_{1}$); when EEs choose ordinary packaging, only basic sales revenue $V_{0}$ is obtained.

When enterprises and consumers have the same understanding of green products and common products, enterprises can better meet the needs of consumers. D’Orazio and Valente, Liao, and Shi found that the public investment banks clearly support green investment, and consistent consumer environmental quality preferences can better stimulate environmental market innovation [49,50]. When the green choices of enterprises and consumers are consistent (enterprises choose green express packages, and consumers join the recycling program; companies use ordinary packaging, and consumers do not join the recycling program), e-commerce companies can more accurately meet the needs of consumers, which will bring an increase in sales volume and lead to more profits (the profits are marked as $Q_{1},Q_{2}$, respectively). The additional utility gains obtained by consumers are $U_{1},U_{2}$, when the green choices of the two are different (which means not both choose green strategy), only basic benefits can be obtained. When ECs and EEs choose the green strategy (join the recycling plan and use express packages), it will have a positive promotional effect on the reputation of e-commerce platforms that have taken green action. From the green action taken, consumers and e-commerce companies can gain $M_{1},M_{2}$, respectively, in turn. When the e-commerce platform does not support and promote green action, the platform gains basic benefits only.

Assumption 3: Sheu and Chen used a triparty game model to point out that the government should use subsidies and green taxation policies to promote the production of green products, thereby promoting the development of green supply chains and related industries [51,52]. The purpose of environmental regulation is to find the best intensity of environmental regulations to coordinate the interests of various stakeholders [53], and the government should take this into account when making decisions [54]. The government can choose to participate in the express package recycling program using different forms of subsidies. Assuming that the total amount of subsidies is S, the share of capital subsidies occupied by e-commerce platforms is $\theta_{1}$, and the share of capital subsidies occupied by consumers is $\theta_{2}$; then, the share of subsidies occupied by e-commerce enterprises is $1-\theta_{1}-\theta_{2}$, of which $\theta_{1},\theta_{2}$ all belong to the range [0,1]. 

### 3.3. Model Solution in the Case of the N-Model

From the above assumptions, we can see that the revenue matrix of the three parties without government subsidies in Table 1. 

The benefits of the e-commerce platform’s selection of support strategies and nonsupport strategies can be marked as $E_{11},E_{12}$, respectively, in order, and their average returns can be marked as $\bar{E_{x1}}$:

$$E_{11}=x_{2}x_{3}({A-C}_{1}+M_{1}+M_{2})+x_{2}(1-x_{3})({A-C}_{1}+M_{1})+(1-x_{2})x_{3}({A-C}_{1}+M_{2})+(1-x_{2})(1-x_{3})({A-C}_{1})$$

Then, the replication dynamic equation of the e-commerce platform is

(1) $$G(x_{1})=\frac{dx_{1}}{dt}=x_{1}(E_{11}-\bar{E_{x1}})=x_{1}(1-x_{1})(E_{11}-E_{12})=A-C_{1}-R_{0}+C_{2}+x_{2}M_{1}+x_{3}M_{2}$$

(2) $$E_{12}=x_{2}x_{3}(R_{0}{-C}_{2})+x_{2}(1-x_{3})(R_{0}{-C}_{2})+(1-x_{2})x_{3}(R_{0}{-C}_{2})+(1-x_{2})(1-x_{3})(R_{0}{-C}_{2})$$

(3) $$\bar{E_{x1}}=x_{1}E_{11}+(1-x_{1})E_{12}$$

Similarly, the benefits of the consumers who choose to join the green recycle plan and not to join the green recycle plan are, respectively, $E_{21},E_{22}$; the average return is $\bar{E_{x2}}$.

(4) $$E_{21}=x_{1}x_{3}(B_{1}{-L}_{1}+D_{1}+U_{1})+x_{1}(1-x_{3})(B_{1}{-L}_{1}+D_{1})+(1-x_{1})x_{3}(B_{1}{-L}_{1}+U_{1})+(1-x_{1})(1-x_{3})(B_{1}{-L}_{1})$$

(5) $$E_{22}=x_{1}x_{3}(B_{0}{-L}_{0})+x_{1}(1-x_{3})(B_{0}{-L}_{0}+U_{2})+(1-x_{1})x_{3}(B_{0}{-L}_{0})+(1-x_{1})(1-x_{3})(B_{0}{-L}_{0}+U_{2})$$

(6) $$\bar{E_{x2}}=x_{2}E_{21}+(1-x_{2})E_{22}$$

Then, the consumer’s replication dynamic equation is

(7) $${G(x}_{2})=\frac{dx_{2}}{dt}=x_{2}(E_{21}-\bar{E_{x2}})=x_{2}(1-x_{2})(E_{21}-E_{22})=B_{1}-L_{1}-B_{0}+L_{0}+x_{1}D_{1}-U_{2}+x_{3}(U_{1}+U_{2})$$

Similarly, it can be seen that the revenue of the e-commerce enterprise’s strategy of selecting ordinary packaging and express packages is $E_{31},E_{32}$, respectively, and the average revenue is $\bar{E_{x3}}$; then,

(8) $$E_{31}=x_{1}x_{2}(V_{1}{-W}_{1}+Q_{1}+D_{2})+x_{1}(1-x_{2})(V_{1}{-W}_{1}+D_{2})+(1-x_{1})x_{2}(V_{1}{-W}_{1}+Q_{1})+(1-x_{1})(1-x_{2})(V_{1}{-W}_{1})$$

(9) $$E_{32}=x_{1}x_{2}(V_{0}{-W}_{2})+x_{1}(1-x_{2})(V_{0}{-W}_{2}+Q_{2})+(1-x_{1})x_{2}(V_{0}{-W}_{2})+(1-x_{1})(1-x_{2})(V_{0}{-W}_{2}+Q_{2})$$

(10) $$\bar{E_{x3}}=x_{3}E_{31}+(1-x_{3})E_{32}$$

Then, the replication dynamic equation of the e-commerce enterprise is

(11) $${G(x}_{3})=\frac{dx_{3}}{dt}=x_{3}(E_{31}-\bar{E_{x3}})=x_{3}(1-x_{3})(E_{31}-E_{32})=V_{1}-W_{1}-V_{0}+W_{2}+x_{1}D_{2}-Q_{2}+x_{2}(Q_{1}+Q_{2})$$

To sum up, the three main parties constitute the evolution system, as follows:

(12) $$\{\begin{array}{l} G(x_{1})=\frac{dx_{1}}{dt}=x_{1}(E_{11}-\bar{E_{x1}})=x_{1}(1-x_{1})(E_{11}-E_{12})=x_{1}(1-x_{1})(A-C_{1}-R_{0}+C_{2}+x_{2}M_{1}+x_{3}M_{2})\\ {G(x}_{2})=\frac{dx_{2}}{dt}=x_{2}(E_{21}-\bar{E_{x2}})=x_{2}(1-x_{2})(E_{21}-E_{22})=x_{2}(1-x_{2})[B_{1}-L_{1}-B_{0}+L_{0}+x_{1}D_{1}-U_{2}+x_{3}(U_{1}+U_{2})]\\ {G(x}_{3})=\frac{dx_{3}}{dt}=x_{3}(E_{31}-\bar{E_{x3}})=x_{3}(1-x_{3})(E_{31}-E_{32})=x_{3}(1-x_{3})[V_{1}-W_{1}-V_{0}+W_{2}+x_{1}D_{2}-Q_{2}+x_{2}(Q_{1}+Q_{2})] \end{array}$$

According to the conclusions of Weibull and Ritzberger and Selten [55,56], let the replication dynamic equation of the above three parties be 0; then, we can obtain the following equilibrium points (0,0,0), (0,1,0), (0,0,1), (1,0,0), (1,1,0), (1,0,1), (0,1,1), (1,1,1). See Appendix A for more details about the local stability judgment of the equilibrium point of the differential equation. According to the Lyapunov method and the stability principle of differential equations [57], the critical conditions for the evolution of each subject can be obtained, which will be described in detail afterward.

#### 3.3.1. E-Commerce Platform Evolution Strategy

**Figure 2:**
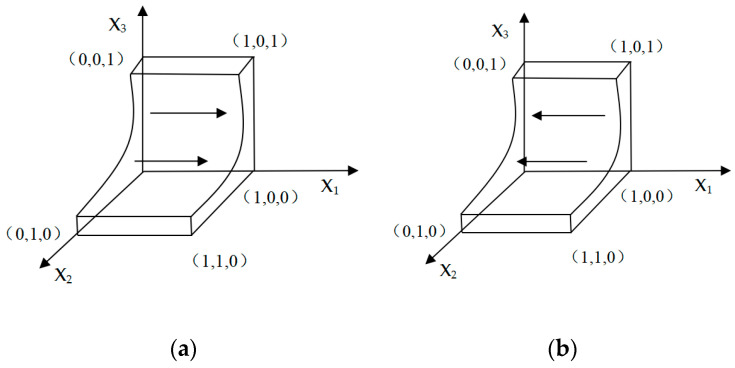
The evolutionary phase diagram of the e-commerce platform.

When $G(x_{1})=0$, we can get the critical point. When $x_{1}(1-x_{1})(A-C_{1}-R_{0}+C_{2}+x_{2}M_{1}+x_{3}M_{2})=0$, we can obtain the critical point at $x_{2}=\frac{C_{1}-R_{0}-A-C_{2}{-x}_{3}M_{2}}{M_{1}}$. When the critical point is obtained, all strategies are evolutionary stable strategies; when $x_{2}>\frac{C_{1}-R_{0}-A-C_{2}{-x}_{3}M_{2}}{M_{1}}$, if $\frac{\partial G(x_{1})}{x_{1}}<0$ is satisfied, $x_{1}>\frac{1}{2}$ needs to be satisfied at this time, which means e-commerce platforms will tend to choose to support the “Green Recycling Program for Express Packaging”. In this situation, the evolution phase diagram of the e-commerce platform is shown in Figure 2a. It can be seen from Figure 2a that at this time x_1_ is evolving away the x_2_ox_3_ side, and e-commerce platforms tend to choose to support the express packaging recycling strategy at this time. When $x_{2}<\frac{C_{1}-R_{0}-A-C_{2}{-x}_{3}M_{2}}{M_{1}}$, if $\frac{\partial G(x_{1})}{x_{1}}<0$ is satisfied, $x_{1}<\frac{1}{2}$ needs to be satisfied at this time, which means e-commerce platforms will tend not to choose to support the “Green Recycling Program for Express Packaging”. In this situation, the evolution phase diagram of the e-commerce platform is shown in Figure 2b. It can be seen from Figure 2b that at this time x_1_ is evolving toward from the x_2_ox_3_ side. At this time, e-commerce platforms tend to choose not to support express packaging recycling strategies.

#### 3.3.2. Consumer Evolutionary Stability

**Figure 3:**
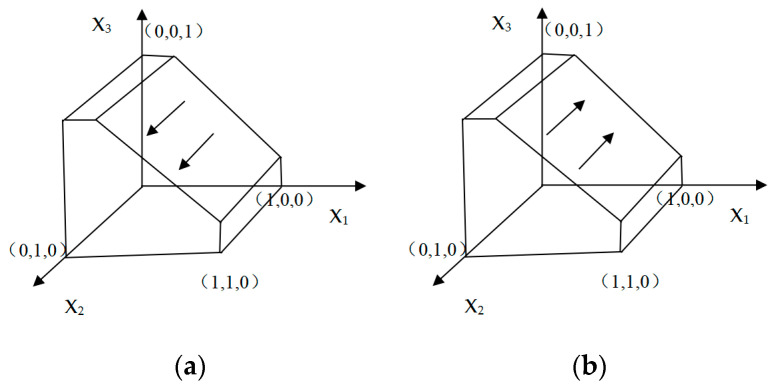
The evolutionary phase diagram of consumers.

Using the same analysis mechanism, when $G(x_{2})=0$, we can obtain $x_{2}(1-x_{2})[B_{1}-L_{1}-B_{0}+L_{0}+x_{1}D_{1}-U_{2}+x_{3}(U_{1}+U_{2})]=0$, and we can get the critical point at $x_{3}=\frac{B_{0}+L_{1}-B_{1}-L_{0}+U_{2}-x_{1}D_{1}}{U_{1}+U_{2}}$. When $x_{3}=\frac{B_{0}+L_{1}-B_{1}-L_{0}+U_{2}-x_{1}D_{1}}{U_{1}+U_{2}}$, all strategies are evolutionarily stable strategies. When $x_{3}>\frac{B_{0}+L_{1}-B_{1}-L_{0}+U_{2}-x_{1}D_{1}}{U_{1}+U_{2}}$, to satisfy $\frac{\partial G(x_{2})}{x_{2}}<0$, $x_{2}>\frac{1}{2}$ needs to be met, which means consumers will tend to choose to join the “Green Recycling Program for Express Packaging”. In this situation, the phase diagram is shown in Figure 3a. It can be seen from Figure 3a that at this time x_2_ is evolving away from the x_1_ox_3_ side. At this time, e-commerce consumers tend to choose to participate in the express packaging recycling strategy. When $x_{3}<\frac{B_{0}+L_{1}-B_{1}-L_{0}+U_{2}-x_{1}D_{1}}{U_{1}+U_{2}}$, to satisfy $\frac{\partial G(x_{2})}{x_{2}}<0$, $x_{2}<\frac{1}{2}$ needs to meet, which means consumers will tend not to choose to join the “Green Recycling Program for Express Packaging”. At this time, the phase diagram is shown in Figure 3b.It can be seen from Figure 3b that at this time x_2_ is evolving towards the x_1_ox_3_ side, and e-commerce consumers tend to choose not to participate in the express packaging recycling strategy.

#### 3.3.3. Evolutionary Stability of E-Commerce Enterprises

**Figure 4:**
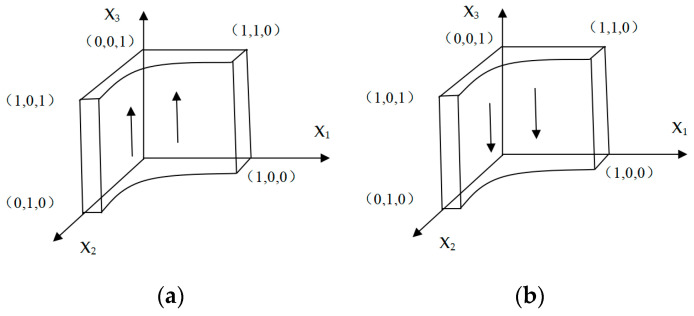
The evolutionary phase diagram of the e-commerce enterprise.

Using the same analysis mechanism, let $G(x_{3})=0$, which is $x_{3}(1-x_{3})[V_{1}-W_{1}-V_{0}+W_{2}+x_{1}D_{2}-Q_{2}+x_{2}(Q_{1}+Q_{2})]=0$; we can get the critical point at $x_{2}=\frac{W_{1}+V_{0}-V_{1}-W_{2}+Q_{2}-x_{1}D_{2}}{Q_{1}+Q_{2}}$. When $x_{2}=\frac{W_{1}+V_{0}-V_{1}-W_{2}+Q_{2}-x_{1}D_{2}}{Q_{1}+Q_{2}}$ is satisfied, all strategies are evolutionarily stable strategies. When $x_{2}>\frac{W_{1}+V_{0}-V_{1}-W_{2}+Q_{2}-x_{1}D_{2}}{Q_{1}+Q_{2}}$, to satisfy $\frac{\partial G(x_{3})}{x_{3}}<0$, $x_{2}>\frac{1}{2}$ needs to be met, which means e-commerce enterprises will tend to choose green packaging. In this situation, the phase diagram is as shown in Figure 4a; It can be seen from Figure 4a that at this time x_3_ is evolving away from the x_1_ox_2_ side. At this time, e-commerce companies tend to choose green express packaging strategies.otherwise, when $x_{2}>\frac{W_{1}+V_{0}-V_{1}-W_{2}+Q_{2}-x_{1}D_{2}}{Q_{1}+Q_{2}}$, to satisfy $\frac{\partial G(x_{3})}{x_{3}}<0$, $x_{2}<\frac{1}{2}$ needs to meet, which means e-commerce enterprises tend to choose ordinary packaging. In this situation, the phase diagram is shown in Figure 4b. It can be seen from Figure 4b that at this time x_3_ is evolving toward the x_1_ox_2_ side, and e-commerce companies tend to choose ordinary express packaging strategies at this time.

#### 3.3.4. Evolutionary Stability of Tripartite Parties

According to Friedman’s conclusion [37], the stability of the differential equation system at the equilibrium point can be analyzed by the Jacobi matrix. The Jacobi matrix to be analyzed in this paper is as follows:

(13) $$\begin{array}{l} J=\left[\begin{array}{ccc} \frac{\partial G(x_{1})}{\partial x_{1}} & \frac{\partial G(x_{1})}{\partial x_{2}} & \frac{\partial G(x_{1})}{\partial x_{3}}\\ \frac{\partial G(x_{2})}{\partial x_{1}} & \frac{\partial G(x_{2})}{\partial x_{2}} & \frac{\partial G(x_{2})}{\partial x_{3}}\\ \frac{\partial G(x_{3})}{\partial x_{1}} & \frac{\partial G(x_{3})}{\partial x_{2}} & \frac{\partial G(x_{3})}{\partial x_{3}} \end{array}\right]=\\ \left[\begin{array}{ccc} \begin{array}{l} (1-2x_{1})(A-C_{1}-R_{0}\\ +C_{2}+x_{2}M_{1}+x_{3}M_{2}) \end{array} & x_{1}(1-x_{1})M_{1} & x_{1}(1-x_{1})M_{2}\\ x_{2}(1-x_{2})D_{1} & \begin{array}{l} (1-2x_{2})[B_{1}-L_{1}-B_{0}+L_{0}\\ +x_{1}D_{1}-U_{2}+x_{3}(U_{1}+U_{2})] \end{array} & x_{2}(1-x_{2})(U_{1}+U_{2})\\ x_{3}(1-x_{3})D_{2} & x_{3}(1-x_{3})(Q_{1}+Q_{2}) & \begin{array}{l} (1-2x_{3})[V_{1}-W_{1}-V_{0}+W_{2}\\ +x_{1}D_{2}-Q_{2}+x_{2}(Q_{1}+Q_{2})] \end{array} \end{array}\right] \end{array}$$

From the above analysis, the characteristic equations and eigenvalues corresponding to the eight pure strategies can be obtained (more details on the process of solving differential equations and calculating characteristic roots are in Appendix A). The eigenvalues are summarized in Table 2.

According to Lyapunov’s method [57], it can be seen that when all the eigenvalues for a strategy are negative, the strategy is locally and progressively stable. By analyzing the eigenvalues corresponding to the pure strategy equilibrium points in the above table, we find that all equilibrium points have the potential to become evolutionary stable points. Therefore, in order to more intuitively and vividly find some management enlightenment, Matlab2016b will be used in the following numerical simulation in Section 3.

### 3.4. Model Solution in the Case of the Y-Model

According to the previous assumption (especially Assumption 3), using a similar analysis mechanism, the evolutionary game matrix that includes government subsidies is shown in Table 3.

According to the same analysis mechanism above, we can obtain the eigenvalues of the replicated dynamic equations; Table 4 is the equilibrium points of each pure strategy in the case of government subsidies (more details on this part can be seen in Appendix B).

(14) $$\{\begin{array}{l} G(x_{1}{}^{\prime })=\frac{dx_{1}}{dt}=x_{1}(1-x_{1})(A-C_{1}-R_{0}+C_{2}+\theta_{1}S+x_{2}M_{1}+x_{3}M_{2})\\ {G(x}_{2}{}^{\prime })=\frac{dx_{2}}{dt}=x_{2}(1-x_{2})[B_{1}-L_{1}-B_{0}+L_{0}+\theta_{2}S+x_{1}D_{1}-U_{2}+x_{3}(U_{1}+U_{2})]\\ {G(x}_{3}{}^{\prime })=\frac{dx_{3}}{dt}=x_{3}(1-x_{3})[V_{1}-W_{1}-V_{0}+W_{2}+(1-\theta_{1}-\theta_{2})S+x_{1}D_{2}-Q_{2}+x_{2}(Q_{1}+Q_{2})] \end{array}$$

For the same analysis mechanism, this paper uses a numerical example simulation to find more management insights; the results in the two cases will be compared later.

## 4. Numerical Simulation

The existing literature [2,6,58] and the actual background of the research problems in this paper are used to set the simulation parameters of this study.

### 4.1. Numerical Simulation of the N-Model (Without Government Subsidies)

**Figure 5:**
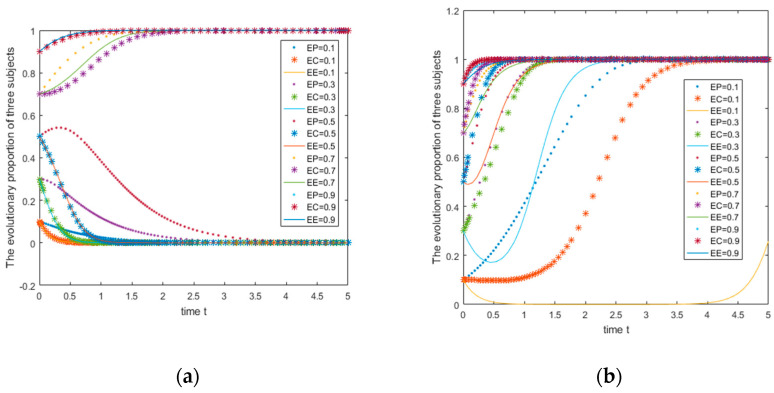
Different strategies correspond to the evolution graph under different basic income scenarios.

When the parameters are $A=5,C_{1}=7,R_{0}=1.5,C_{2}=2,M_{1}=2,M_{2}=3,B_{1}=5,L_{1}=8,B_{0}=1,L_{0}=2,D_{1}=2,$ $D_{2}=4,U_{1}=3,U_{2}=5,V_{1}=3,V_{0}=5,W_{1}=5,W_{2}=4,Q_{1}=3,Q_{2}=5$, the initial probabilities are 0.1, 0.3, 0.5, 0.7, and 0.9, respectively. In this situation, basic revenue parameters [59] and action consistency parameters are all small; the numerical simulation image is shown in Figure 5a. It can be seen from Figure 5a that with the increase of the initial probabilities of the three players, the strategy choices of the three players change from (not supported, not participating, ordinary) to a combination of strategies (supporting, participating, green).

When the parameters are $A=7,C_{1}=5,R_{0}=2,C_{2}=1.5,M_{1}=3,M_{2}=2,B_{1}=8,L_{1}=5,B_{0}=2,L_{0}=1,D_{1}=4,$ $D_{2}=2,U_{1}=5,U_{2}=3,V_{1}=3,V_{0}=5,W_{1}=5,W_{2}=4,Q_{1}=5,Q_{2}=3$, the initial probabilities are 0.1, 0.3, 0.5, 0.7, and 0.9. In this situation, the basic revenue parameters and the action consistency parameters are all large; the numerical simulation image is shown in Figure 5b. It can be seen from Figure 5b that no matter how the initial probability changes, the strategy combination of the three will not change, and it is always stable at the strategy combination (support, participate, green).

**Figure 6:**
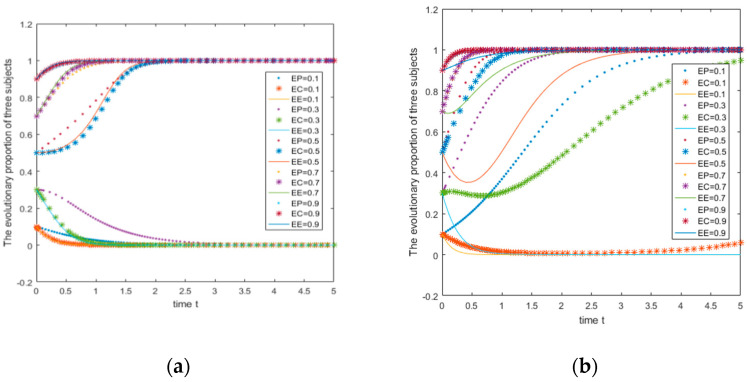
Different strategies correspond to the evolution graph under different basic income scenarios (the benefit of consistent action is the same).

It can be seen from Figure 5b that the size of the basic income of each party choosing different strategies has a significant impact on the evolution of the actor’s strategy. When the basic income is positive, that is, when the net income increases, the actor can quickly converge to the strategy (1,1,1), and this result is not affected by the initial probability. When the basic income is negative (see Figure 5a), that is to say, when net income decreases, the evolution direction of the subject is related to the initial probability: when the initial probability is 0.1, 0.3 and 0.5, the system will converge to the strategy (0,0,0), and when the initial probability is 0.7 and 0.9, the system will converge to (1,1,1). According to the relevant information of People’s Daily, the current recovery rate of Chinese express packaging is still relatively low. Therefore, it is necessary to identify some key factors to promote the evolution of the subject towards (1,1,1) and provide suggestions to the relevant decision-makers. In this case, this article chooses for the action consistency parameter and the basic return parameter direction to be consistent. This leads to the question of what kind of results the system will evolve towards when the parameter directions of the two are inconsistent. Figure 6 shows the research and answers this question.

When the parameters are $L_{0}=2,D_{1}=2,D_{2}=4,U_{1}=5,U_{2}=3,V_{1}=3,V_{0}=5,W_{1}=5,W_{2}=4,Q_{1}=5,Q_{2}=3$, $L_{0}=2,D_{1}=2,D_{2}=4,U_{1}=5,U_{2}=3,V_{1}=3,V_{0}=5,W_{1}=5,W_{2}=4,Q_{1}=5,Q_{2}=3$, the initial probabilities are 0.1, 0.3, 0.5, 0.7, and 0.9. In this situation, the basic revenue parameters are small and the action consistent parameters are large; the numerical simulation image is shown in Figure 6a. It can be seen from Figure 6a that as the initial probabilities of the three agents increase, the three players’ strategic choices change from (not supported, not participating, ordinary) to a combination of strategies (supporting, participating, green). The evolution is basically similar to Figure 5a.

When the parameters are $A=7,C_{1}=5,R_{0}=2,C_{2}=1.5,M_{1}=3,M_{2}=2,B_{1}=8,L_{1}=5,B_{0}=2,L_{0}=1,D_{1}=4,D_{2}=2,U_{1}=3,U_{2}=5,V_{1}=3,V_{0}=5,$ $W_{1}=5,W_{2}=4,Q_{1}=3,Q_{2}=5$, the initial probabilities are 0.1, 0.3, 0.5, 0.7, and 0.9. In this situation, the basic revenue parameters are all large, and the consistent action parameters are all small; the numerical simulation image is shown in Figure 6b. It can be seen from Figure 6b that with the increase of the initial probabilities of the three entities at this time, the strategies of the e-commerce platform will not change, and they are all supporting strategies. However, the strategies of consumers and e-commerce companies will gradually change from (not participating, ordinary) to (participating, green).

From Figure 6a, we can see that the evolution of the system when the parameter directions of basic return and consistent action return are different. When basic income has a net decrease and consistent action income has a net increase, the evolution of the image is basically the same as Figure 5a. When basic income has a net increase and action consistent income has a net decrease, the evolution image (Figure 6b) is different from Figure 5b. When the net income from the concerted action has a net increase, it means ($U_{1}{>U}_{2}{,Q}_{1}{>Q}_{2}$), that is to say, consumers and e-commerce companies have a stronger awareness of green environmental protection, and both parties have a higher enthusiasm to participate in express package recycling activities. Higher income, on the contrary, indicates that the two have insufficient awareness of green environmental protection and are less active in participating in express package recycling. The comparison of Figure 5b and Figure 6b shows that when there is a net increase in basic income and a net decrease in consistent action income, consumers may still converge to 0 at a lower initial probability of 0.1 and 0.3, and e-commerce companies converge to 0 at a lower initial probability of 0.1, which shows that when the basic benefit of the green action strategy is large, the system may still be the result of an undesirable “non-green” strategy because of the inconsistent action of the two parties. Therefore, it is still necessary to further discuss the impact of the different changes of the two types of income on the equilibrium evolution results.

#### The Initial Probability Remains Unchanged, and the Consistent Parameter Returns

**Figure 7:**
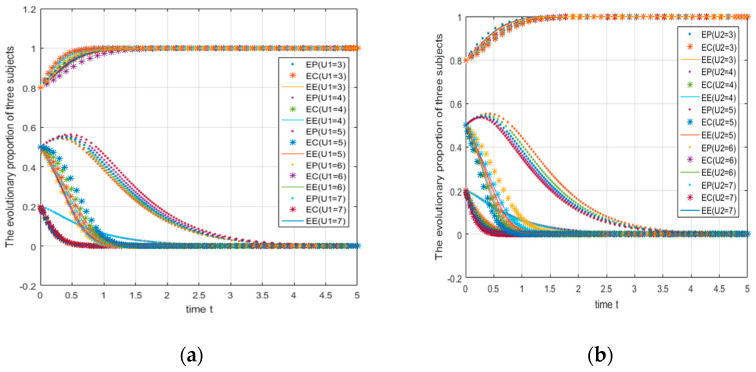
Evolutionary image when only the parameters of consumers’ consistent behavior change.

The parameters are $A=5,C_{1}=7,R_{0}=1.5,C_{2}=2,M_{1}=2,M_{2}=3,$ $B_{1}=5,L_{1}=8,B_{0}=1,L_{0}=2,D_{1}=2,$$D_{2}=4,U_{1}=3-7,U_{2}=5,V_{1}=3,V_{0}=5,W_{1}=5,W_{2}=4,Q_{1}=3,Q_{2}=5$. In this situation, the basic income parameters are all small and the initial probabilities are 0.2, 0.5, and 0.8. The numerical simulation image is shown in Figure 7a. It can be seen from Figure 7a that as the benefits of consumers increase when their actions of consumers and e-commerce companies are inconsistent, the three strategic choices change from (not supporting, not participating, ordinary) to a combination of strategies (supporting, participating, green )change. Moreover, the evolution result will also be affected by the initial selection probability.

The parameters are $A=5,C_{1}=7,R_{0}=1.5,C_{2}=2,M_{1}=2,M_{2}=3,B_{1}=5,L_{1}=8,B_{0}=1,L_{0}=2,D_{1}=2,D_{2}=4,$ $U_{1}=3,U_{2}=3-7,V_{1}=3,V_{0}=5,W_{1}=5,W_{2}=4,Q_{1}=3,Q_{2}=5$. In this situation, the basic income parameters are all small, $U_{2}=3-7$, and the initial probabilities are 0.2, 0.5, and 0.8. The numerical simulation image is shown in Figure 7b. It can be seen from Figure 7b that the evolution at this time is basically similar with Figure 7a, and the explanation will not be repeated redundantly here.

It can be found from the above figure that the evolutionary images of the three parties are basically unchanged when the consumer’s behavior is consistent with the change of revenue parameter. When one of the parameters ($U_{1},U_{2}$) changes from 3 to 7, the impact on the final evolution result is very weak.

**Figure 8:**
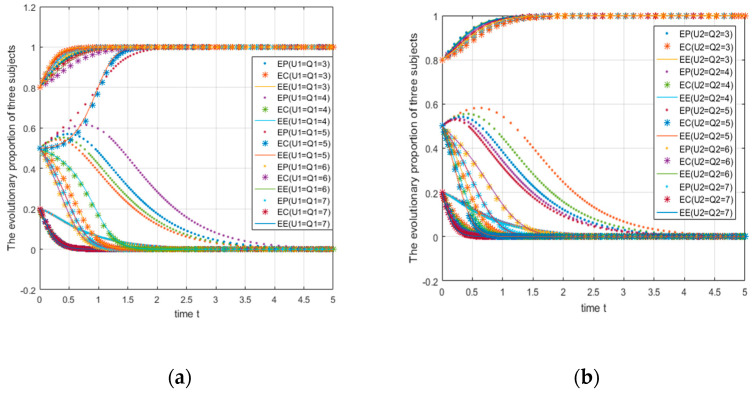
The evolutionary image of consumers and e-commerce companies with consistent revenue parameters while changing in the same direction.

Take the parameters as $A=5,C_{1}=7,R_{0}=1.5,C_{2}=2,M_{1}=2,M_{2}=3,B_{1}=5,L_{1}=8,B_{0}=1,L_{0}=2,D_{1}=2,$ $D_{2}=4,U_{1}=3-7,U_{2}=5,V_{1}=3,V_{0}=5,W_{1}=5,W_{2}=4,Q_{1}=3-7,Q_{2}=5$. In this situation, the basic income parameters are all small, and the initial probabilities are 0.2, 0.5, and 0.8. The numerical simulation image is shown in Figure 8a. It can be seen from Figure 8a that when the synergistic benefits of consumers and e-commerce companies change in the same direction and proportion, as the benefits of the two choose green strategies (participation, green) increase, the strategy evolution shows a more complicated change at an initial probability of 0.5.

Take the parameters as $A=5,C_{1}=7,R_{0}=1.5,C_{2}=2,M_{1}=2,M_{2}=3,B_{1}=5,L_{1}=8,B_{0}=1,L_{0}=2,D_{1}=2,$ $D_{2}=4,U_{1}=3,U_{2}=3-7,V_{1}=3,V_{0}=5,W_{1}=5,W_{2}=4,Q_{1}=3,Q_{2}=3-7$. In this situation, the basic income parameters are all small, and the initial probabilities are 0.2, 0.5, and 0.8. The numerical simulation image is shown in Figure 8b. It can be seen from Figure 8b that when the synergistic benefits increase in the same proportion and in the same direction, the revenue of the two choose non-green strategies (no participation, ordinary) increase, their strategy choices are only affected by the initial selection probability.

It can be seen from Figure 8b that when $U_{2}$ and $Q_{2}$ change together in the same direction, when the initial probability is 0.2 and 0.5, the three parties of the system will eventually evolve toward (0,0,0); when the initial probability is at 0.8, the system eventually evolves towards the strategy (1,1,1). When $U_{1}$ and $Q_{1}$ change together, and the initial probability is 0.5, it presents a more complicated evolutionary result (see Figure 8a). It can be found that when the initial probability of the three parties is 0.5, when $U_{1}=Q_{1}=5$, the three subject strategies converge to (1,1,1), and when $U_{1}$ and $Q_{1}$ are equal, to 3, 4, 6, and 7. The three subject strategies converge to (0,0,0), which shows that the green action’s consistent return, being too high or too low, is not beneficial to the evolution of the equilibrium towards the ideal situation (1,1,1).

### 4.2. Simulation of Numerical Examples for the Y-Model (with Government Subsidies) 

#### 4.2.1. Impact of Changes in Subsidy Ratio Coefficient on System Evolution

**Figure 9:**
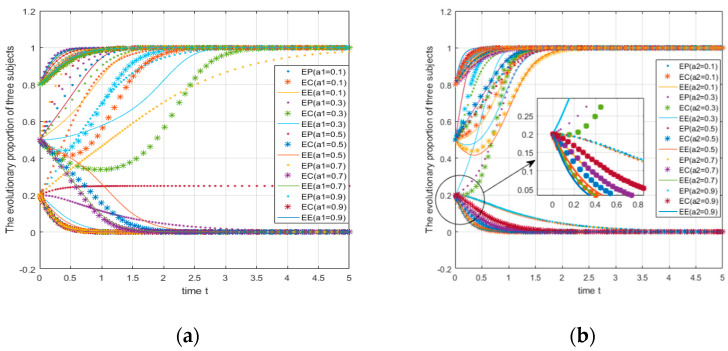
Impact of changes in the subsidy’s proportional coefficient on system evolution.

Take the parameters as $A=5,C_{1}=7,R_{0}=1.5,C_{2}=2,M_{1}=2,M_{2}=3,B_{1}=5,L_{1}=8,B_{0}=1,L_{0}=2,D_{1}=2,$$D_{2}=4,U_{1}=3,U_{2}=5,V_{1}=3,V_{0}=5,W_{1}=5;W_{2}=4,Q_{1}=3,Q_{2}=5,S=5,\theta_{2}=0.1;\theta_{1}=a_{1}=\left\{0.1,0.3,0.5,0.7,0.9\right\}$; the initial probabilities are 0.2, 0.5, and 0.8. In this situation, the numerical simulation image is shown in Figure 9a. It can be seen from Figure 9a that as the share of government subsidies to e-commerce platforms gradually increases, when the initial probability of the three entities choosing “green strategies” is relatively low (0.2, 0.5), the incentive effect on e-commerce platforms is more obvious. When the initial probability increases to a higher level, the subsidy loses its incentive effect.

Take the parameters as $A=5,C_{1}=7,R_{0}=1.5,C_{2}=2,M_{1}=2,M_{2}=3,B_{1}=5,L_{1}=8,B_{0}=1,L_{0}=2,D_{1}=2,$ $D_{2}=4,U_{1}=3,U_{2}=5,V_{1}=3,V_{0}=5,W_{1}=5;\;W_{2}=4,Q_{1}=3,Q_{2}=5,S=5,\theta_{2}=0.1,\theta_{1}=\left\{0.1,0.3,0.5,0.7,0.9\right\}$; the initial probabilities are 0.2, 0.5, and 0.8. In this situation, the numerical simulation image is shown in Figure 9b. It can be seen from Figure 9b that as the share of government subsidies to consumers gradually increases, the incentive effect of consumer subsidies will only play a significant role when the initial probability of the three subjects choosing the “green strategy” is extremely low (0.2).

It can be seen from Figure 9a that the consumer (EC) accounts for the subsidy ratio of 0.1 at this time. When the initial probability of the three parties is 0.2, e-commerce companies and consumers quickly converge to strategy 0; in other words, the two ultimately choose to use ordinary packages and not join the express package recycling program. When e-commerce platforms account for the subsidy ratio of 0.3 (the proportion of e-commerce companies at this time is 0.6), the e-commerce platform evolves towards strategy 0. When the subsidy ratio of e-commerce platforms is 0.5 (at this time, the proportion of e-commerce companies is 0.4), the strategy is basically the same. When the initial probability is 0.5, the evolution of the e-commerce platform is not affected by the change of the subsidy coefficient and can quickly converge to 1. When the e-commerce platform subsidy coefficient is 0.1, 0.3, and 0.5, the subsidy coefficients of e-commerce companies should be 0.8, 0.6, and 0.4 to facilitate its evolution to Strategy 1. Therefore, when the government subsidizes the three parties, the proportion of e-commerce enterprises cannot be too low (the threshold is between 0.2–0.4); otherwise, it will not work to achieve an ideal state. 

When the e-commerce platform’s fixed subsidy ratio remains unchanged at 0.1 and the consumer’s subsidy ratio changes (see Figure 9b), it can be found that when the initial probability of the three parties are 0.5 and 0.8, the system will move toward (1,1,1). When the initial probability is 0.2, only when the consumer’s subsidy coefficient is 0.2 (at this time, the e-commerce enterprise’s subsidy coefficient is 0.7) can the system finally converge to (1,1,1), which means that when the awareness of green environmental protection is relatively low (that is, the probability of the three parties choosing green actions is relatively low), the government should mainly subsidize e-commerce enterprises. Combined with the previous discussion and the analysis of the subsidy coefficient of the e-commerce platform, we can draw a conclusion: the government’s subsidy should focus on e-commerce enterprises, which is more beneficial to achieving the ideal goal.

#### 4.2.2. Impact of Changes in Subsidy Amount on System Evolution

**Figure 10:**
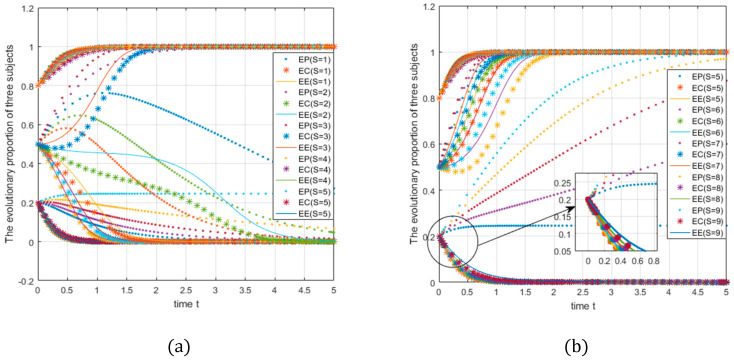

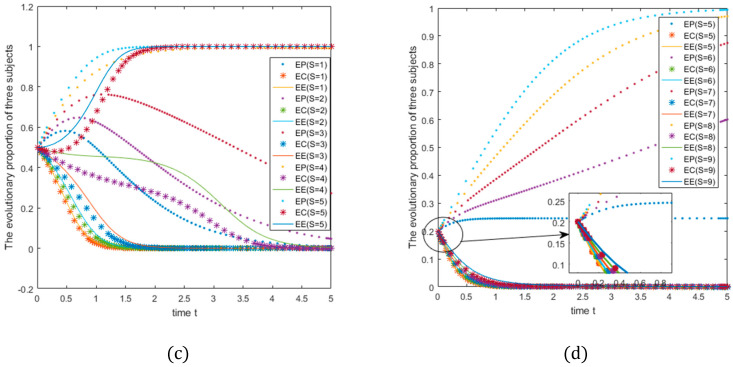
Impact of changes in the amount of subsidies on system evolution.

Take the parameters as $A=5,C_{1}=7,R_{0}=1.5,C_{2}=2,M_{1}=2,M_{2}=3,B_{1}=5,L_{1}=8,B_{0}=1,L_{0}=2,D_{1}=2,$ $D_{2}=4,U_{1}=3,U_{2}=5,V_{1}=3,V_{0}=5,W_{1}=5,W_{2}=4,Q_{1}=3,Q_{2}=5,\theta_{2}=0.3,\theta_{1}=0.3,S=1-9$; the initial probabilities are 0.2, 0.5, and 0.8. Graph (a) of Figure 10 shows the parameter S changing from 1 to 5. Graph (b) of Figure 10 shows the parameter S changing from 5 to 9, and, to show more details, Graphs (c), (d) of Figure 10 show the evolution at the initial probabilities of 0.2 and 0.5 alone (which means the images in the second row are part of the first row). 

At this time, the e-commerce platform’s fixed subsidy coefficient and the consumer subsidy coefficient is 0.3, and the e-commerce enterprise subsidy coefficient is 0.4. When the total amount of subsidies changes from 1 to 5, the initial probability is fixed at 0.5 (see Figure 10a). For e-commerce platforms, as the total amount of subsidies increases, the strategy converges from the beginning at 0 (S = 1,2,3) to 1 (S = 4,5), In other words, the threshold value of the transition is between 3–4, and there are similar conclusions for e-commerce companies and consumers. The critical value of strategic change is higher (between 4–5) for e-commerce platforms and consumers. In summary, it can be found that the evolution of the three strategies is closely related to the total subsidy amount, and consumers and e-commerce companies need higher subsidy amounts to achieve ideal state evolution. 

With an initial probability of 0.2, as the total subsidy amount changes from 5 to 9 (see Figure 10b), both consumers and e-commerce companies converge to 0, while e-commerce platforms converge to 1 at an increasing rate. This shows that preferential subsidies to e-commerce platforms in the case of low initial probability should be given priority; at this time, the subsidies have a weaker incentive for consumers and e-commerce companies to choose green strategies; hence, they need to be paid more subsidy. When the initial probability is as high as the medium level of 0.5, the total amount of subsidies that encourages the three to choose green strategies (support, join, green) is relatively low, and the threshold is between 3–5. Overall, subsidies have a strong incentive effect on e-commerce platforms. In addition, government subsidies can “correct” the high-cost locking phenomenon of nongreen strategies to a certain extent, which also shows us that in the process of implementing the express package recycling program, the incentive effect of government subsidies is crucial [53,54]. The Chinese government’s environmental regulation through administrative intervention and certain incentives is effective and realistic [60].

## 5. Discussion and Recommendations

### 5.1. Discussion of Results

With the vigorous development of e-commerce, express package waste problems have been generated in the process of online shopping, which has caused harm to the environment and ecology. The Chinese government is cooperating with large e-commerce platforms to realize the recycling of express packages. In reality, e-commerce companies can freely choose whether to use recyclable packaging for express packages, and consumers can choose whether to join this activity. For e-commerce companies, the cost of choosing packaging is an important factor that needs to be considered when making decisions. The foundation of this article shows that the basic benefits of actors choosing different strategies count. If the net benefit of choosing one strategy is increased, then this action can easily persist and be promoted. Many studies have confirmed that the effect of basic income parameters on equilibrium evolution is extremely significant [2]. For consumers participating in express package recycling, costs, including time and money costs, need to be considered. However, for consumers with strong environmental awareness, they can get a certain sense of satisfaction in the process of participating. If consumers like to participate in the recycling process of express packages, those e-commerce companies that choose to use green package materials are more likely to be selected. Of course, consumers who do not like to participate can also choose those that use ordinary packaging. The online merchants make a choice. These two types of merchants and consumers mean that e-commerce companies and consumers have to produce a kind of synergy. When strategies diverge, there is only basic shopping income between the two. The view of “seeing what you get or lose under different strategies” is like the two aspects of a coin, which does not affect the results of qualitative analysis. As the conclusions in the article reveal, the synergistic benefits of consumers and e-commerce companies can “correct” the impact of basic benefits to a certain extent, which is easy to understand. In the case of the cost-saving consideration of basic benefits, e-commerce companies and consumers are more inclined to choose not to participate and ordinary packages; it is precisely the existence of synergistic benefits that makes it possible to participate in express package recycling. In this process, consumers get greater net benefits (such as the compensation effect of psychological satisfaction). For e-commerce companies, the use of recyclable and green express packages can also have increased net benefits (such as the reputation of the e-commerce companies) when there are synergistic benefits. As pointed out in many economic studies, goodness and altruistic behaviors are common in people’s behaviors [61], which is also one of the reasons why “synergistic benefits” exist.

For a newly promulgated environmental policy, it often takes a certain amount of time to be accepted by the public. The government’s administrative guidance can shorten the time to achieving this goal. In the past, the promotion of environmental policies related to land and energy received relevant government policy support and subsidy incentives [6]. The recycling of express packages is a new issue that has recently emerged. Although many e-commerce platforms have responded to some extent after the introduction of relevant policies, the practice is still not mature. As a result, the participation of merchants and consumers on the platform is not enough, as many environmental regulation documents have pointed out [61]. Merchants on many platforms, including JD.com and Taobao, still mainly use corrugated boxes and plastic packaging materials; the recycling rate is very low. Damania et al. has advised on increasing the cost of choosing “nonideal” strategies (in his original manuscript, the corruption costs of enterprises and local governments) [62], which is not applicable to issues such as express packages. On the one hand, the use of ordinary packaging materials cost less for e-commerce companies, and we cannot change this. However, considering the existence of environmentally-friendly consumers and government subsidies, e-commerce companies will weigh the pros and cons when making decisions. To a certain extent, subsidies can also play the role of an incentive, thereby affecting behavioral decisions [44]. The conclusion of this article points out that when the initial probability of the three choices (support, participation, and green) is relatively low, the subsidies should be given mainly to e-commerce companies, and governments should focus on e-commerce platforms. This finding is in line with intuition and reality. First of all, when the initial probability is relatively low, related issues such as express package waste would have just appeared; the public’s awareness is insufficient, and the understanding of express packaging mixed waste is insufficient. At this time, more publicity and promotion work is needed. In e-commerce transactions, e-commerce merchants and consumers are “closer”; they can communicate directly and clarify each other’s needs. At this time, giving priority to subsidizing e-commerce merchants is a natural and effective decision. When the public’s awareness of this issue reaches a certain level, e-commerce companies, consumers, and e-commerce platforms will be more enthusiastic about participating in express packaging recycling. As we know, the bilateral market characteristics [63] of e-commerce platforms can serve as a hub and link connecting logistics and information flow, and e-commerce companies and consumers can communicate on it. At this time, the emphasis on subsidizing e-commerce platforms to encourage them to improve their coordination and scheduling role is obviously more conducive to the evolution of the equilibrium state toward the ideal goal (support, join, green). The finding in this article has important practical significance for relevant policymakers to determine the priority of subsidies.

### 5.2. Case Study

**Figure 11:**
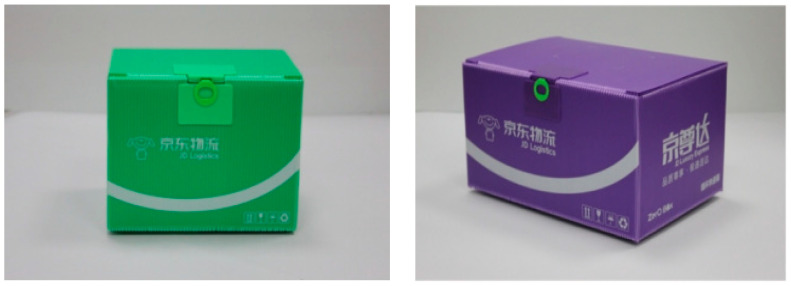
Image of a “Qingliu” box from JD.com.

This section uses JD.com ‘s express packaging recycling practices to explain the case. There are two reasons: on the one hand, JD.com has a self-operated online sales system; hence, it can be regarded as an e-commerce company. In addition, it can also accept other merchants as an e-commerce platform. Moreover, it has also built its own logistics system for its own use or used by merchants on its platform. JD.com ’s practices are representative and demonstrative. Moreover, as the top first-responder of the express package recycling program of the corresponding China Post Bureau, JD.com’s actions are relatively advanced and exploratory. The theoretical models in this paper have produced some valuable enlightenment to guide policymakers. When the China Post Bureau pointed out the plans for express package recycling, JD.com responded in a timely fashion to this call and implemented a series of express package recycling programs. In 2016, on the day of the Mid-Autumn Festival (a traditional Chinese festival; online transaction volumes near the date increase heavily, and the quantity of packaging materials will increase simultaneously), JD.com launched the “Carton Recycling, Green and Environmental Protection” project, which won widespread respect in the community. In March 2017, the carton recycling plan was further upgraded: consumers could exchange discarded carton (picked up by JD.com express couriers) for a certain amount of Jingdou tokens (a virtual token of the JD.com platform) to receive discounts on JD.com’s products). At first, this activity was only carried out in Beijing, Shanghai, Guangzhou, and Shenzhen. The recovered carton would be further recycled through JD.com’s warehouse logistics system. Follow-up practices are likely to be further promoted in other cities in China. In addition to carton recycling, JD.com also launched a customized version of a better-quality express box called the “Qingliu” box, shown in Figure 11. This is a reusable logistics box made from new resin materials.

On 7 December 2017, JD.com officially launched the product called “green box”, with the first batch of 100,000 units. This green and clean box is made of a new type of thermoplastic resin material, with a hollow plate structure, which can be shaped and packaged in five seconds. It has the advantages of anti-shock and high and low temperature/humidity resistance. It can be reused more than 20 times and can be quickly decomposed after being retired. In the entire recycling system, JD.com has adopted ways of cooperating with third parties to operate the entire process of recycling, disinfection, cleaning, and remanufacturing of the logistics boxes. JD.com’s open logistics packaging circulation cooperation system can be regarded as a “footnote” in JD.com’s building block theory in the state of “unbound retail”. Although long-term value requires a certain amount of time to be observed, these practices still provide some reference and inspiration as to the successor to express package recycling. On 5 June 2017, JD.com’s logistics enterprise signed a joint action “Green Flow Plan” for a green supply chain with nine major manufacturers, including Procter & Gamble, Nestlé, Lego, Kimberly-Clark, Nongfu Spring, Watsons, Unilever, and Yili. Plus, JD.com also signed the “China Paper Product Sustainability Initiative” with the environmental protection organization WWF (World Wildlife Fund) to help green express delivery and packaging recycling.

In addition to the efforts of e-commerce platforms and e-commerce companies for express package recycling, government officials have also made suggestions as to these issues. CPPCC member (representatives of government officials who can discuss political issues and give advice to the government) Yan Zuoting proposed that the government should take the lead in implementing the send-and-fetch mechanism for express packages, the coupon exchange mechanism, and certain subsidy or reward policies for the environmental-friendly actions that are needed. Additionally, logistics recycling companies can co-build express package recycling systems. At the same time, logistics companies should also be encouraged to develop and adopt more environmental-friendly, green, and recyclable materials for express packaging. 

Some of the practices of other countries regarding the recycling of packaging are also worth learning; the practices are summarized in Table 5. The United States cut taxes for packaging companies that recycle and passed the “Resource Protection and Recycling Act” for industrial cooperation to carry out packaging recycling. Some companies have led the establishment of a carton council. Japan legislated the “Packaging Recycling and Reuse Law” for package reuse and established a certain number of recycling sites. After consumers sort the waste, the specialized personnel will regularly supervise and recycle. Germany has issued the “Packaging Waste Management Measures”, which propose that package waste should be processed in the order of reduction–reuse–recycling–final disposal and specified the recycling standards and targets. It is mandatory for package manufacturers and sellers to be jointly responsible for package recycling so that every link of packaging processing has quantitative standards for reference. France directly stipulates in packaging waste transportation law that consumers are obliged to give package waste to retailers and manufacturers for processing; the government has set up waste recycling institutions, with manufacturers as shareholders. Moreover, guarantor institutions will supervise the process. The Netherlands has developed an “ecotax”, which can be reduced by using paper packaging and other recyclable packaging materials. The use of other materials is taxed, and the process of reusing packaging requires records as evidence. The Dutch packaging industry representative signed a contract with the government. The contract stipulates that more than 65% of package materials must be reusable, of which 45% of package materials must be recycled and 20% of the materials require incineration to produce energy. From Table 5, we find that many countries have adopted legislation and taxation to promote the recycling of package waste. Moreover, the process and other specific regulations and constraints on package materials also should be taken into account; these measures have certain reference and value to policymakers in China’s express packaging recycling programs.

## 6. Conclusions

With the development of e-commerce businesses, various materials are used in the packaging process of express packages, and a large amount of packaging waste has not been reasonably treated and reused. The Chinese government is cooperating with large e-commerce platforms to realize the recycling of express packaging. In reality, e-commerce companies can freely choose whether to use recyclable green packaging for express packaging, and consumers can choose whether to join in this activity. If the two parties reach a tacit agreement on this “green decision”, there will be synergy. In addition, the existing literature on environmental regulation has shown that the necessary subsidies are effective when implementing a policy that is beneficial to the environment and ecology [62]. This study uses two triparty evolutionary game models to study the strategic choices and evolution of consumers, e-commerce companies, and e-commerce platforms on the issue of express packaging recycling. At the same time, the model considers whether there are government subsidies.

The study found that the decision of whether the three entities participate in express packaging recycling is significantly affected by basic income, where the existence of synergistic income and government subsidies can “affect” the lock-in state of basic income on the equilibrium result. In the case of the Y-model (with government subsidies), when the probability of participating in express delivery is low (early stage), the subsidy should be focused on e-commerce companies. When the three entities’ willingness to participate gradually increases to a high level, the government should mainly subsidize the electricity platform. These results have provided us with some recommendations: first, e-commerce companies can conduct demand surveys for different commodities and obtain consumer acceptance of recyclable express packages and gradually promote this express package recycling program among different types of goods. Propaganda activities such as green express packaging lectures and raising the public’s awareness of green express packaging will help improve the overall national ecological civilization concept. In this way, the ecological civilization of the entire population will be improved, and consumers and businesses will have a higher motivation to choose to support and join green express packaging recycling. Only by mobilizing the actions of the population can it be possible to achieve the goal of building a green recycling system for express packaging as soon as possible. Secondly, we can learn from the practice of JD.com; the platform provides certain virtual rewards to consumers who actively participate in express packaging (for example, JD.com provides certain Jingdou rewards to consumers who participate in express package recycling). As far as the relevant government subsidies are concerned, public awareness of the recycling of express packaging is not very strong in the current stage; it is still in an early stage. Therefore, subsidies should be focused on e-commerce enterprises. For example, certain financial support, tax reductions, or official commendations can be used to reduce their costs and improve their corporate reputations. These actions can be regarded as rewards and subsidies for e-commerce companies. When public participation in express package recycling has gradually increased to a certain stage, the government should focus on subsidizing e-commerce platforms. At this time, e-commerce platforms should effectively use their central and coordinating role to build a smooth information flow channel for consumers, businesses, and logistics. Close cooperation between various subjects can better achieve this ideal evolutionary goal.

Of course, there are still some limitations in this article. First of all, there are many factors that affect the choice of different stakeholders in express packaging recycling. This article only selects some factors for discussion. The issue of express package waste has recently attracted widespread attention and discussion, but the time that has passed is too short. It is difficult to obtain effective real data. Random parameters have been chosen in the simulation, which is one of the limitations of this article. Secondly, whether it supports multipurpose packaging and whether the packaging is recyclable varied in different contexts. In the future, the economic impact of express packages can be studied from the perspective of the packaging’s life cycle and supply chain. In addition, packaging recycling cannot “naturally occur” and requires the participation of actors (like consumers, policymakers, online merchants). Considering the preferences and attitudes of participants is an interesting research extension. Moreover, more abundant and diverse data can be collected to provide more solid and instructive empirical evidence.

## Figures and Tables

**Table 1:** Game revenue matrix without government participation.

Strategy Mix	Electronic Business Platform	Consumer	E-Commerce Enterprise
(EP_2_, EC_2_, EE_2_)	$R_{0}{-C}_{2}$	$B_{0}{-L}_{0}+U_{2}$	$V_{0}{-W}_{2}+Q_{2}$
(EP_2_, EC_2_, EE_1_)	$R_{0}{-C}_{2}$	$B_{0}{-L}_{0}$	$V_{1}{-W}_{1}$
(EP_2_, EC_1_, EE_2_)	$R_{0}{-C}_{2}$	$B_{1}{-L}_{1}$	$V_{0}{-W}_{2}$
(EP_1_, EC_2_, EE_2_)	${A-C}_{1}$	$B_{0}{-L}_{0}+U_{2}$	$V_{0}{-W}_{2}+Q_{2}$
(EP_1_, EC_1_, EE_2_)	${A-C}_{1}+M_{1}$	$B_{1}{-L}_{1}+D_{1}$	$V_{0}{-W}_{2}$
(EP_1_, EC_2_, EE_1_)	${A-C}_{1}+M_{2}$	$B_{0}{-L}_{0}$	$V_{1}{-W}_{1}+D_{2}$
(EP_2_, EC_1_, EE_1_)	$R_{0}{-C}_{2}$	$B_{1}{-L}_{1}+U_{1}$	$V_{1}{-W}_{1}+Q_{1}$
(EP_1_, EC_1_, EE_1_)	${A-C}_{1}+M_{1}+M_{2}$	$B_{1}{-L}_{1}+D_{1}+U_{1}$	$V_{1}{-W}_{1}+Q_{1}+D_{2}$

**Table 2:** The characteristic roots corresponding to the equilibrium point without government subsidies.

Equilibrium Point	The Characteristic Root Corresponding to the Equilibrium Point
(0,0,0)	$A-C_{1}-R_{0}+C_{2},B_{1}-L_{1}-B_{0}+L_{0}-U_{2},V_{1}-W_{1}-V_{0}+W_{2}-Q_{2}$
(0,1,0)	$A-C_{1}-R_{0}+C_{2}+M_{1},-(B_{1}-L_{1}-B_{0}+L_{0}-U_{2}),V_{1}-W_{1}-V_{0}+W_{2}+Q_{1}$
(1,0,0)	$-(A-C_{1}-R_{0}+C_{2}),B_{1}-L_{1}-B_{0}+L_{0}-U_{2}+D_{1},V_{1}-W_{1}-V_{0}+W_{2}+D_{2}-Q_{2}$
(0,0,1)	$A-C_{1}-R_{0}+C_{2}+M_{2},B_{1}-L_{1}-B_{0}+L_{0}+U_{1},-(V_{1}-W_{1}-V_{0}+W_{2}-Q_{2})$
(1,1,0)	$-(A-C_{1}-R_{0}+C_{2}+M_{1}),-(B_{1}-L_{1}-B_{0}+L_{0}+D_{1}-U_{2}),V_{1}-W_{1}-V_{0}+W_{2}+D_{2}-Q_{1}$
(1,0,1)	$-(A-C_{1}-R_{0}+C_{2}+M_{2}),B_{1}-L_{1}-B_{0}+L_{0}+D_{1}+U_{1},-(V_{1}-W_{1}-V_{0}+W_{2}+D_{2}-Q_{2})$
(0,1,1)	$A-C_{1}-R_{0}+C_{2}+M_{1}+M_{2},-(B_{1}-L_{1}-B_{0}+L_{0}+U_{1}),-(V_{1}-W_{1}-V_{0}+W_{2}+Q_{1})$
(1,1,1)	$-(A-C_{1}-R_{0}+C_{2}+M_{1}+M_{2}),-(B_{1}-L_{1}-B_{0}+L_{0}+D_{1}+U_{1}),-(V_{1}-W_{1}-V_{0}+W_{2}-D_{2}+Q_{1})$

**Table 3:** Game revenue matrix with government participation.

Strategy Mix	Electronic Business Platform	Consumer	E-Commerce Enterprise
(EP_2_,EC_2_,EE_2_)	$R_{0}{-C}_{2}$	$B_{0}{-L}_{0}+U_{2}$	$V_{0}{-W}_{2}+Q_{2}$
(EP_2_,EC_2_,EE_1_)	$R_{0}{-C}_{2}$	$B_{0}{-L}_{0}$	$V_{1}{-W}_{1}+(1-\theta_{1}-\theta_{2})S$
(EP_2_,EC_1_,EE_2_)	$R_{0}{-C}_{2}$	$B_{1}{-L}_{1}+\theta_{2}S$	$V_{0}{-W}_{2}$
(EP_1_,EC_2_,EE_2_)	${A-C}_{1}+\theta_{1}S$	$B_{0}{-L}_{0}+U_{2}$	$V_{0}{-W}_{2}+Q_{2}$
(EP_1_,EC_1_,EE_2_)	${A-C}_{1}+M_{1}+\theta_{1}S$	$B_{1}{-L}_{1}+D_{1}+\theta_{2}S$	$V_{0}{-W}_{2}$
(EP_1_,EC_2_,EE_1_)	${A-C}_{1}+M_{2}+\theta_{1}S$	$B_{0}{-L}_{0}$	$V_{1}{-W}_{1}+D_{2}+(1-\theta_{1}-\theta_{2})S$
(EP_2_,EC_1_,EE_1_)	$R_{0}{-C}_{2}$	$B_{1}{-L}_{1}+U_{1}+\theta_{2}S$	$V_{1}{-W}_{1}+Q_{1}+(1-\theta_{1}-\theta_{2})S$
(EP_1_,EC_1_,EE_1_)	${A-C}_{1}+M_{1}+M_{2}+\theta_{1}S$	$B_{1}{-L}_{1}+D_{1}+U_{1}+\theta_{2}S$	$V_{1}{-W}_{1}+Q_{1}+D_{2}+(1-\theta_{1}-\theta_{2})S$

**Table 4:** Equilibrium points and corresponding characteristic roots with government subsidies.

Equilibrium Point	The Characteristic Root Corresponding to the Equilibrium Point
(0,0,0)	$A-C_{1}-R_{0}+C_{2},B_{1}-L_{1}-B_{0}+L_{0}-U_{2},V_{1}-W_{1}-V_{0}+W_{2}-Q_{2}$
(0,1,0)	$A-C_{1}-R_{0}+C_{2}+M_{1},-(B_{1}-L_{1}-B_{0}+L_{0}-U_{2}),V_{1}-W_{1}-V_{0}+W_{2}+Q_{1}$
(1,0,0)	$-(A-C_{1}-R_{0}+C_{2}),B_{1}-L_{1}-B_{0}+L_{0}-U_{2}+D_{1},V_{1}-W_{1}-V_{0}+W_{2}+D_{2}-Q_{2}$
(0,0,1)	$A-C_{1}-R_{0}+C_{2}+M_{2},B_{1}-L_{1}-B_{0}+L_{0}+U_{1},-(V_{1}-W_{1}-V_{0}+W_{2}-Q_{2})$
(1,1,0)	$-(A-C_{1}-R_{0}+C_{2}+M_{1}),-(B_{1}-L_{1}-B_{0}+L_{0}+D_{1}-U_{2}),V_{1}-W_{1}-V_{0}+W_{2}+D_{2}-Q_{1}$
(1,0,1)	$-(A-C_{1}-R_{0}+C_{2}+M_{2}),B_{1}-L_{1}-B_{0}+L_{0}+D_{1}+U_{1},-(V_{1}-W_{1}-V_{0}+W_{2}+D_{2}-Q_{2})$
(0,1,1)	$A-C_{1}-R_{0}+C_{2}+M_{1}+M_{2},-(B_{1}-L_{1}-B_{0}+L_{0}+U_{1}),-(V_{1}-W_{1}-V_{0}+W_{2}+Q_{1})$
(1,1,1)	$-(A-C_{1}-R_{0}+C_{2}+M_{1}+M_{2}),-(B_{1}-L_{1}-B_{0}+L_{0}+D_{1}+U_{1}),-(V_{1}-W_{1}-V_{0}+W_{2}-D_{2}+Q_{1})$

**Table 5:** Summary of packaging recycling practices in different countries.

Country	Legislation	Taxation	Cooperating with the Government	Building Special Agency	Requirements for the Quantity of Recovery
United States	◎	◎		◎	
Japan	◎			◎	
Germany	◎				◎
France	◎			◎	
Netherlands		◎			◎
Belgium			◎	◎	◎

Note: The symbol “◎” indicates the existence of such measures.

## Appendix A

Take the pure strategy equilibrium point (0,0,0) in the N-model as an example (other solving processes are similar and will not be repeated here):

Bring this point into the Jacobian matrix of the N-model:

(A1) $$J_{\left(0,0,0\right)}=\left[\begin{array}{ccc} A-C_{1}-R_{0}+C_{2} & & \\ & B_{1}-L_{1}-B_{0}+L_{0}-U_{2} & \\ & & V_{1}-W_{1}-V_{0}+W_{2}-Q_{2} \end{array}\right]$$

The characteristic roots of the above matrix $J_{\left(0,0,0\right)}$ are

(A2) $$\lambda_{1}=A-C_{1}-R_{0}+C_{2},\lambda_{2}=B_{1}-L_{1}-B_{0}+L_{0}-U_{2},\lambda_{3}=V_{1}-W_{1}-V_{0}+W_{2}-Q_{2}$$

According to the judgment criterion (Lyapunov method), when $\lambda_{1}<0,\lambda_{2}<0,\lambda_{3}<0$, (0,0,0) is a locally stable point; otherwise, it is a saddle point (Haiyan Shan and Junliang Yang (2019)).

## Appendix B. Construction of an Evolutionary Game Model with Government Subsidies (Y-Model)

According to the same analysis mechanism as above, we can get the expected return ($E_{11}{}^{\prime },E_{12}{}^{\prime },E_{21}{}^{\prime },E_{22}{}^{\prime },E_{31}{}^{\prime },E_{32}{}^{\prime }$) and average expected return ($\bar{E_{x1}{}^{\prime }},\bar{E_{x2}}{}^{\prime },\bar{E_{x3}}{}^{\prime }$) of the e-commerce platform, consumers and e-commerce enterprises, in the case of government subsidies, when choosing different strategies:

(A3) $$E_{11}{}^{\prime }=x_{2}x_{3}({A-C}_{1}+M_{1}+M_{2}+\theta_{1}S)+x_{2}(1-x_{3})({A-C}_{1}+M_{1}+\theta_{1}S)+(1-x_{2})x_{3}({A-C}_{1}+M_{2}+\theta_{1}S)+(1-x_{2})(1-x_{3})({A-C}_{1}+\theta_{1}S)$$

(A4) $$E_{12}{}^{\prime }=x_{2}x_{3}(R_{0}{-C}_{2})+x_{2}(1-x_{3})(R_{0}{-C}_{2})+(1-x_{2})x_{3}(R_{0}{-C}_{2})+(1-x_{2})(1-x_{3})(R_{0}{-C}_{2})$$

(A5) $$\bar{E_{x1}}{}^{\prime }=x_{1}E_{11}+(1-x_{1})E_{12}$$

(A6) $$\begin{array}{l} E_{21}{}^{\prime }=x_{1}x_{3}(B_{1}{-L}_{1}+D_{1}+U_{1}+\theta_{2}S)+x_{1}(1-x_{3})(B_{1}{-L}_{1}+D_{1}+\theta_{2}S)+\\ \left(1-x_{1})x_{3}(B_{1}{-L}_{1}+U_{1}+\theta_{2}S)+(1-x_{1})(1-x_{3})(B_{1}{-L}_{1}+\theta_{2}S\right) \end{array}$$

(A7) $$E_{22}{}^{\prime }=x_{1}x_{3}(B_{0}{-L}_{0})+x_{1}(1-x_{3})(B_{0}{-L}_{0}+U_{2})+(1-x_{1})x_{3}(B_{0}{-L}_{0})+(1-x_{1})(1-x_{3})(B_{0}{-L}_{0}+U_{2})$$

(A8) $$\begin{array}{l} E_{31}{}^{\prime }=x_{1}x_{2}(V_{1}{-W}_{1}+Q_{1}+D_{2}+(1-\theta_{1}-\theta_{2})S)+x_{1}(1-x_{2})[V_{1}{-W}_{1}+D_{2}\\ +(1-\theta_{1}-\theta_{2})S]+(1-x_{1})x_{2}[V_{1}{-W}_{1}+Q_{1}+(1-\theta_{1}-\theta_{2})S]+(1-x_{1})(1-x_{2})[V_{1}{-W}_{1}+(1-\theta_{1}-\theta_{2})S] \end{array}$$

(A9) $$E_{32}{}^{\prime }=x_{1}x_{2}(V_{0}{-W}_{2})+x_{1}(1-x_{2})(V_{0}{-W}_{2}+Q_{2})+(1-x_{1})x_{2}(V_{0}{-W}_{2})+(1-x_{1})(1-x_{2})(V_{0}{-W}_{2}+Q_{2})$$

(A10) $$\bar{E_{x3}}{}^{\prime }=x_{3}E_{31}+(1-x_{3})E_{32}$$

According to the same analysis mechanism as the previous section, the evolutionary equation system of the three parties, with government subsidies, is as follows:

(A11) $$\{\begin{array}{l} G(x_{1}{}^{\prime })=\frac{dx_{1}}{dt}=x_{1}(1-x_{1})(A-C_{1}-R_{0}+C_{2}+\theta_{1}S+x_{2}M_{1}+x_{3}M_{2})\\ {G(x}_{2}{}^{\prime })=\frac{dx_{2}}{dt}=x_{2}(1-x_{2})[B_{1}-L_{1}-B_{0}+L_{0}+\theta_{2}S+x_{1}D_{1}-U_{2}+x_{3}(U_{1}+U_{2})]\\ {G(x}_{3}{}^{\prime })=\frac{dx_{3}}{dt}=x_{3}(1-x_{3})[V_{1}-W_{1}-V_{0}+W_{2}+(1-\theta_{1}-\theta_{2})S+x_{1}D_{2}-Q_{2}+x_{2}(Q_{1}+Q_{2})] \end{array}$$

In this situation, the Jacobian matrix corresponding to the system is

(A12) $$J=\left[\begin{array}{ccc} \begin{array}{l} (1-2x_{1})(A-C_{1}-R_{0}\\ +C_{2}+x_{2}M_{1}+x_{3}M_{2}+\theta_{1}S) \end{array} & x_{1}(1-x_{1})M_{1} & x_{1}(1-x_{1})M_{2}\\ x_{2}(1-x_{2})D_{1} & \begin{array}{l} (1-2x_{2})[B_{1}-L_{1}-B_{0}+L_{0}\\ +x_{1}D_{1}-U_{2}+x_{3}(U_{1}+U_{2})+\theta_{2}S] \end{array} & x_{2}(1-x_{2})(U_{1}+U_{2})\\ x_{3}(1-x_{3})D_{2} & x_{3}(1-x_{3})(Q_{1}+Q_{2}) & \begin{array}{l} (1-2x_{3})[V_{1}-W_{1}-V_{0}+W_{2}+x_{1}D_{2}\\ -Q_{2}+x_{2}(Q_{1}+Q_{2})+(1-\theta_{1}-\theta_{2})S] \end{array} \end{array}\right]$$
